# Integrated analysis of disulfidptosis-related immune genes signature to boost the efficacy of prognostic prediction in gastric cancer

**DOI:** 10.1186/s12935-024-03294-5

**Published:** 2024-03-25

**Authors:** Jie Li, Tian Yu, Juan Sun, Mingwei Ma, Zicheng Zheng, Yixuan He, Weiming Kang, Xin Ye

**Affiliations:** Department of General Surgery, Peking Union Medical College Hospital, Chinese Academy of Medical Sciences & Peking Union Medical College, No.1 Shuaifu Yuan, Dongcheng District, Beijing, 100730 Republic of China

**Keywords:** Gastric cancer, Disulfidptosis, Immune infiltration, Prognostic signature, Single-cell RNA sequencing

## Abstract

**Background:**

Gastric cancer (GC) remains a malignant tumor with high morbidity and mortality, accounting for approximately 1,080,000 diagnosed cases and 770,000 deaths worldwide annually. Disulfidptosis, characterized by the stress-induced abnormal accumulation of disulfide, is a recently identified form of programmed cell death. Substantial studies have demonstrated the significant influence of immune clearance on tumor progression. Therefore, we aimed to explore the intrinsic correlations between disulfidptosis and immune-related genes (IRGs) in GC, as well as the potential value of disulfidptosis-related immune genes (DRIGs) as biomarkers.

**Methods:**

This study incorporated the single-cell RNA sequencing (scRNA-seq) dataset GSE183904 and transcriptome RNA sequencing of GC from the TCGA database. Disulfidptosis-related genes (DRGs) and IRGs were derived from the representative literature on both cell disulfidptosis and immunity. The expression and distribution of DRGs were investigated at the single-cell level in different GC cell types. Pearson correlation analysis was used to identify the IRGs closely related to disulfidptosis. The prognostic signature of DRIGs was established using Cox and LASSO analyses. We then analyzed and evaluated the differences in long-term prognosis, Gene Set Enrichment Analysis (GSEA), immune infiltration, mutation profile, *CD274* expression, and response to chemotherapeutic drugs between the two groups. A tissue array containing 63 paired GC specimens was used to verify the expression of 4 DRIGs and disulfidptosis regulator *SLC7A11* through immunohistochemistry staining.

**Results:**

The scRNA-seq analysis found that *SLC7A11*, *SLC3A2*, *RPN1* and *NCKAP1* were enriched in specific cell types and closely related to immune infiltration. Four DIRGs (*GLA*, *HIF-1α*, *VPS35* and *CDC37*) were successfully identified to establish a signature to potently predict the survival time of GC patients. Patients with high risk scores generally experienced worse prognoses and exhibited greater resistant to classical chemotherapy drugs. Furthermore, the expression of *GLA*, *HIF-1α*, *VPS35*, *CDC37* and *SLC7A11* were elevated in GC tissues. A high expression of *GLA*, *HIF-1α*, *VPS35* or *CDC37* was associated with more advanced clinical stage of GC and increased *SLC7A11* expression.

**Conclusion:**

Current study first highlights the potential value of DRIGs as biomarkers in GC. We successfully constructed a robust model incorporating four DRIGs to accurately predict the survival time and clinicopathological characteristics of GC patients.

**Supplementary Information:**

The online version contains supplementary material available at 10.1186/s12935-024-03294-5.

## Introduction

Gastric cancer (GC) has consistently placed among the top five tumor burden worldwide, with the number of new cases and deaths increasing over the past two decades [1–6]. Due to poor dietary habits and *Helicobacter pylori* infection, China accounts for more than 40% of the global incidence and mortality of GC annually [7, 8]. Consequently, accurate prognosis prediction and management play a crucial role in addressing this issue, in addition to enhancing comprehensive patient treatments [9]. Tumor-node-metastasis (TNM) staging is a widely used tool in clinical practice for predicting prognosis and guiding postoperative treatment for GC patients [10]. Multiple studies have demonstrated that multi-gene signatures with TNM staging can effectively improve the accuracy of prognosis prediction and enable more precise postoperative therapies for patients [11–13]. Therefore, it is necessary to develop an effective signature that can forecast the survival time and risk stratification of GC patients, thus reducing the burden of GC in China.

Normal cell apoptosis is crucial for the development of organisms as it maintains the stability of the microenvironment [14]. However, aberrant cell death can significantly impact tumor progression [15–17]. Under glucose deprivation, cells with high levels of *SLC7A11* consumed excessive amounts of NADPH, resulting in the accumulation of abnormal disulfides, such as cystine. This ultimately induces cell death caused by disulfide stress-induced actin cytoskeleton protein disulfide bond cross-linking, cytoskeleton contraction, and stripping from the plasma membrane. This newly identified mode of programmed cell death is known as disulfidptosis [18]. Immune evasion in the tumor microenvironment (TME) is regulated by a variety of immune-related genes (IRGs), cytokines, chemokines, and metabolites [19]. This necessitated the use of immune checkpoint inhibitors (ICIs) as part of the comprehensive treatment for GC patients. On the other hand, disulfidptosis of tumor cells may have a certain relationship to the immune response in TME. The signal molecules or metabolites generated by disulfidptosis of tumor cells may enhance the recognition of antigen-presenting cells, thereby triggering the immune clearance of CD8^+^ T cells and boosting cellular immunity. However, the intrinsic correlation between disulfidptosis and immune response in GC remains largely unknown. Hence, we aim to explore the relationship between disulfidptosis-related genes (DRGs) and IRGs through bioinformatics analysis. Moreover, the establishment of disulfidptosis-related immune genes (DRIGs) signature is critical for risk stratification and developing individualized treatment strategies for GC patients.

In this study, we examined the expression and distribution of four DRGs at single-cell level in GC. Then, four DIRGs (*GLA*, *HIF-1α*, *VPS35* and *CDC37*) were successfully identified to construct a signature that potent predictive value for the prognosis of GC patients. The intrinsic regulatory network between *GLA*, *HIF-1α*, *VPS35*, *CDC37* and disulfidptosis was further elaborated in GC. In addition, *GLA*, *HIF-1α*, *VPS35*, *CDC37* and the key regulator of disulfidptosis, *SLC7A11*, were detected in the tissue array of GC. The constructed signature using these four DRIGs accurately predicted the survival time and clinicopathological characteristics of GC patients.

## Materials and methods

### Tissue specimen

The tissue array containing 63 paired GC tissues and the corresponding adjacent tissues was gathered from the Department of General Surgery, Peking Union Medical College Hospital (PUMCH) from September 2021 to December 2022. The dissected tissues were fully soaked and fixed in liquid formaldehyde and embedded in paraffin within one week to ensure long-term preservation and subsequent immunohistochemical staining. Prior to tissue sample collection, all patients provided informed consent. This project was approved by the Ethics Committee of the PUMCH (Reference number: K1447).

### Data collection

The single-cell RNA sequencing (scRNA-seq) dataset GSE183904 was obtained from the GEO database [20]. All 18 GC samples from this dataset were included. The TCGA data containing the TPM (Transcripts Per Million) profile, count data matrix, somatic mutation data, survival data and clinical information of GC patients were acquired from UCSC Xena (https://xenabrowser.net/datapages/) [21]. The data was re-annotated using with Gene Symbol before further analysis using the annotation file (gencode.v22.annotation.gene.probeMap). Subsequently, we extracted the clinical data of the corresponding patients and retained GC patients with complete prognostic information to establish a prognostic signature. Finally, the GSE62254 dataset containing 300 samples and the intact clinical information was used to validate the accuracy of the disulfidptosis-related immune genes signature in the prognostic prediction of GC patients [22].

### Quality control, cluster analysis and cell type annotation of scRNA-seq

The detailed steps of quality control of scRNA-seq are as follows: (1) The Seurat object for this analysis was created by using the Seurat (version 4.0) R package imported the GSE183904 [20]. Single-cell data filtering was performed using the following criteria: cells were retained if they had a number of detected RNA features (nFeature_RNA) greater than 100 and less than 5000, as well as a percentage of mitochondrial genes (percent.mito) less than 20%. Cells meeting these criteria (nFeature_RNA > 100 & nFeature_RNA < 5000 & percent.mito < 20) were included in the subsequent analysis. In total, 83,371 cells met these filtering criteria and were used for further analysis. (2) scRNA-seq dataset is standardized by the ‘NormalizeData’ function. (3) Variable counts were determined by invoking the ‘FindVariableFeatures’ function, which identified 3000 variable counts in the dataset. (4) The data was then scaled using the ‘ScaleData’ function and principal component analysis (PCA) was performed to identify significant principal components. The ‘ElbowPlot’ function with variable genes as input was used to determine the top 20 principal components, which were selected for the subsequent uniform manifold approximation and projection (UMAP) analysis (dims = 20). The ‘FindClusters’ function is used for cell clustering. Additionally, the ScType software was utilized to annotate cell types and identify differential marker genes between cell populations [23]. The ‘FindAllMarkers’ function was used to compare the gene expression between different cell types using the Wilcoxon rank-sum test to identify differential genes between cell types.

### KEGG and GO enrichment analysis

KEGG is a widely used database for storing data on genomes, biological pathways, diseases and drugs [24]. Gene Ontology (GO) enrichment is also employed to investigate differentially expressed DIRGs from large-scale functional enrichment at levels of biological process, molecular function and cellular component [25]. *p* value < 0.05 was considered statistically significant, and the enrichment results are further visualized using bubble plots.

### Construction of a prognostic signature

In our research focused on gastric cancer samples with complete survival data, we developed a prognostic model using gene expression profiles. Employing a combination of analytical methods including univariate Cox regression, the Least Absolute Shrinkage and Selection Operator (LASSO), and multivariate Cox regression analysis, we identified significant genes from a set of DIRGs. This meticulous approach allowed us to construct a prognostic model for patients with gastric cancer. The calculation of the risk score, a key component of our model, proceeded as follows:

$$riskScore={\sum }_{i}^{n}Coef\left(gen{e}_{i}\right) *Expression \left(gen{e}_{i}\right)$$

Coef (gene_i_), expression (gene_i_) and n represent the coefficient, the expression of each gene and the number of genes, respectively.

### Construction of a predictive nomogram

The integration of risk scores and clinicopathological characteristics was accomplished using the RMS package (version 5.1-4), facilitating the creation of a nomogram and calibration curve. This calibration curve served to assess the congruence between predicted survival probabilities and actual outcomes, with the ideal predictive accuracy depicted by a 45° line.Following this, the survival package was employed to construct a forest plot, enabling a visual examination of the influence exerted by each clinicopathological feature and risk score on the prognosis.

### Estimation of immune cell infiltration

CIBERSORTx is an analytical tool designed to assess the infiltration of immune cells [26]. The estimation of presumed immune cell abundance was carried out through a reference dataset comprising 22 immune cell subtypes, utilizing 1000 permutations for accuracy. This process, when paired with the LM22 characteristic gene matrix, allowed for the filtration of samples exhibiting a p-value of less than 0.05, thereby acquiring the immune cell infiltration matrix. Subsequently, only data showcasing an immune cell enrichment fraction above zero were preserved to finalize the immune cell infiltration matrix. Pearson correlation analysis was then applied to explore the association between the infiltrating immune cells and genes within the disulfideptosis-related immune gene signature.

### Construction of an online tool for survival probability of GC patients

A web server featuring a dynamic nomogram was developed to predict the survival probability of GC patients. This interactive tool was created using the “DynNom” and “Shiny” packages in R, leveraging the capabilities of the Shiny web platform (https://www.shinyapps.io/). Comprehensive user manuals detailing the server’s operation, output result interpretation, and application of findings to specific scenarios are available in Supplementary File S1. 

### Analysis of somatic mutations and chemosensitivity

The somatic mutation data of TCGA-STAD were acquired utilizing the ‘TCGAbiolinks’ R package [27]. Subsequently, the data were formatted into a Mutation Annotation Format (MAF) file and examined using the ‘maftools’ R package. The analysis of chemosensitivity was conducted in accordance with the methodology outlined in an earlier study [11].

### Immunohistochemistry (IHC)

The procedures of conducting IHC were consistent with the previous publications [28, 29]. The primary antibodies used for the IHC staining were *VPS35* (Abcam, ab157220), *HIF-1α* (Abcam, ab92498), *GLA* (Abcam, ab168341), *CDC37* (Abcam, ab108305) and *SLC7A11* (Abcam, ab175186). The dilution ratio of each primary antibody and the detailed condition of the corresponding antigen repair were under the manufacturer’s instructions. Two random fields of each tissue sample were observed under the 40× objective lens. Then, Image-Pro Plus software was used to assess the expression of each protein using the integrated optical density (IOD) method consisting of staining area and intensity. The final IOD value for each tissue sample was obtained by summing the IOD values from the two random fields.

### Statistics analysis

All data calculations and statistical analyses were performed using R (version 4.1). Statistical significance for the analysis of transcriptome RNA sequencing and scRNA-seq data was set at *p* value < 0.05 or adj. *p* value < 0.05.

## Results

### Expression and mutation profile of disulfidptosis-related genes (DRGs) in GC

**Fig. 1:**
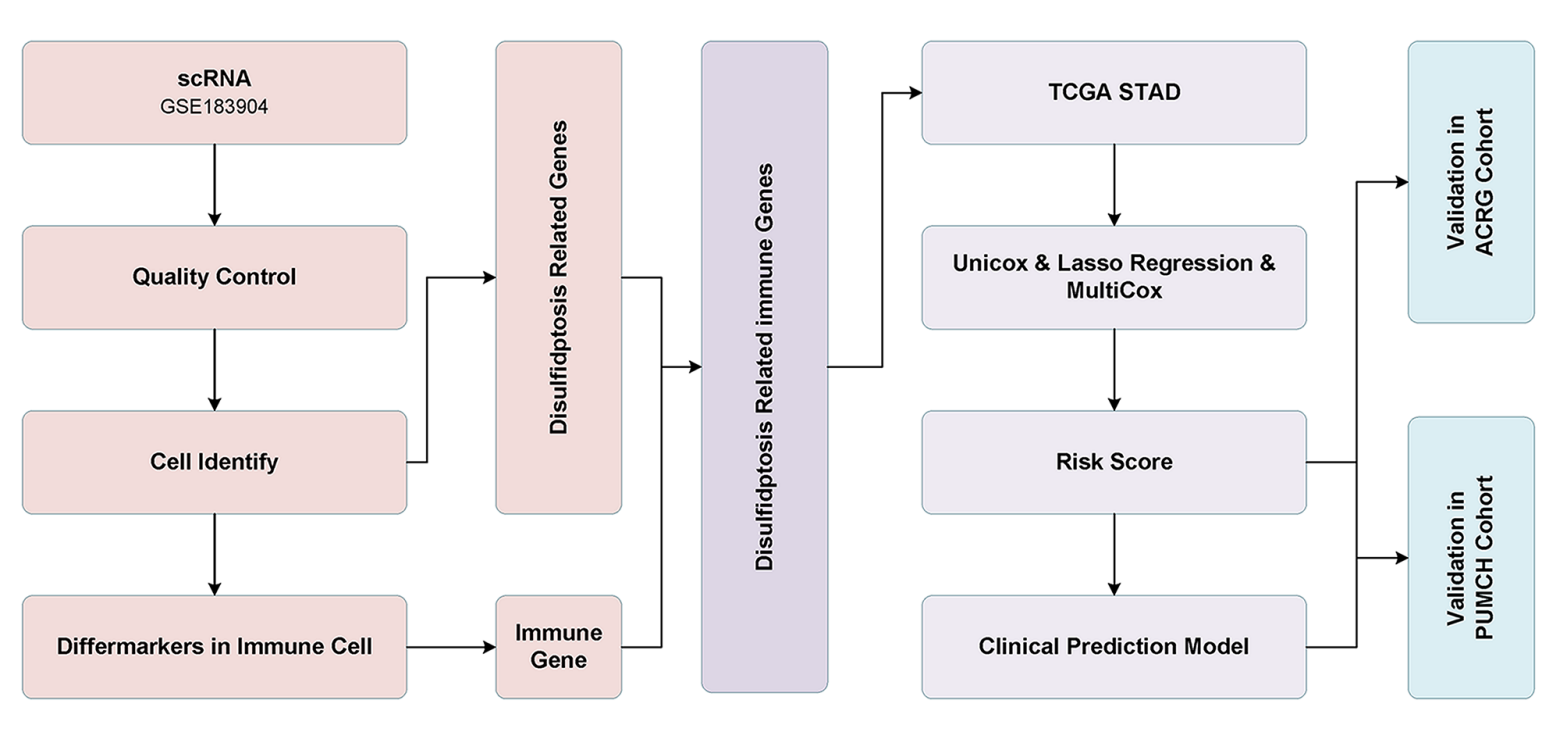
The analysis flow of this study

**Fig. 2:**
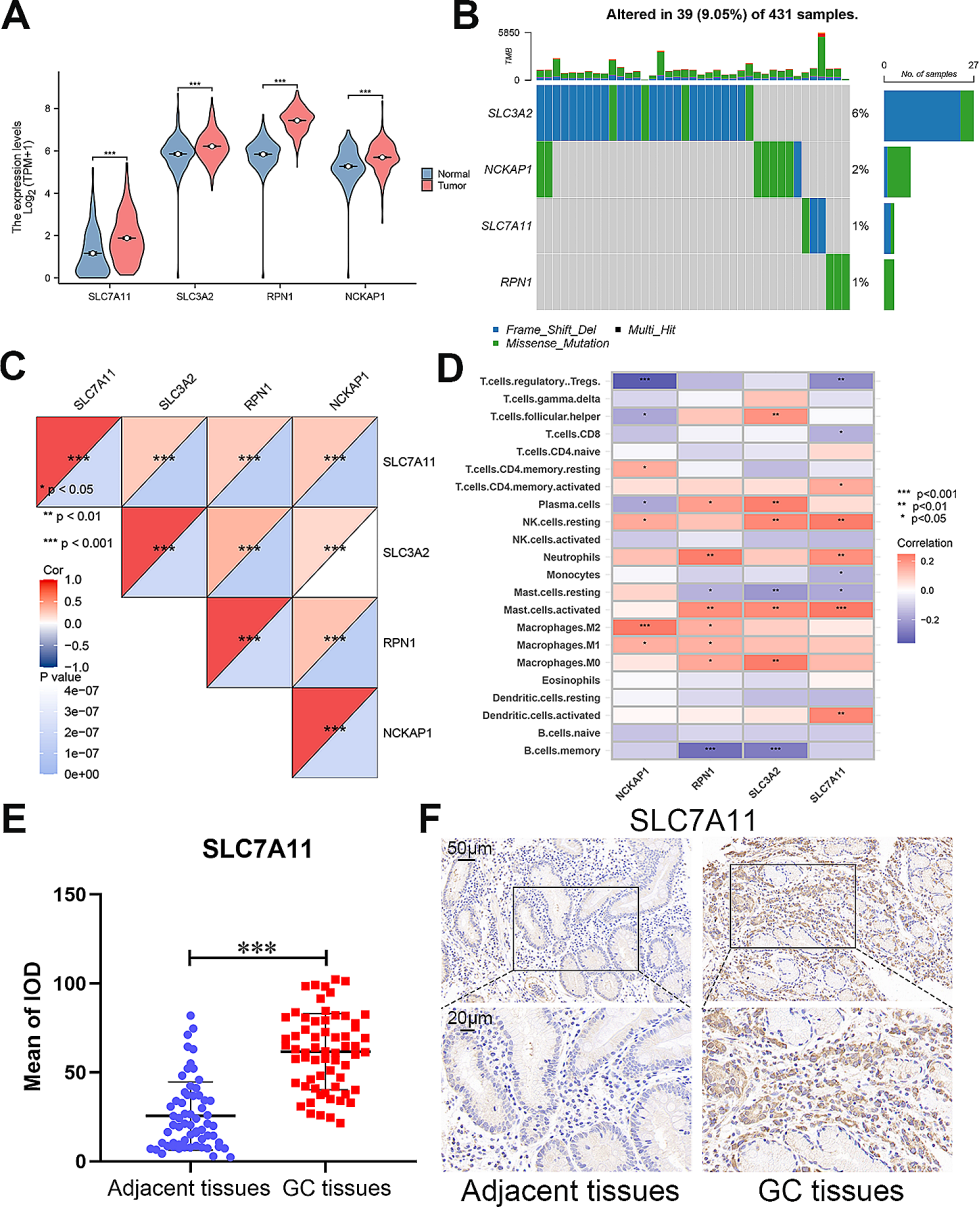
The expression of disulfidptosis-related genes in gastric cancer (GC). **A** Violin plots of SLC7A11, SLC3A2, RPN1 and NCKAP1 expression in GC and adjacent normal tissues from the TCGA. **B** Mutation profile of SLC7A11, SLC3A2, RPN1 and NCKAP1 in 431 GC patients from the TCGA. **C** Correlation of SLC7A11, SLC3A2, RPN1 and NCKAP1 expression. **D** Correlation between SLC7A11, SLC3A2, RPN1 and NCKAP1 and immune cell infiltration. **E** The expression of SLC7A11 in 63 paired GC and adjacent normal tissues. **F** The IHC of SLC7A11 in GC and adjacent normal tissues (scale bar: 50 μm and 20 μm). Student’s t-test was used to determine statistical significance: ***p < 0.001

The flow chart listed the detailed procedures to excavate the values of DRIGs in this study, based on quality control and identification of cell subtypes using single-cell data from GC, analyzing the characterization of immune cell-specific genes within the GC microenvironment, correlating them with DRGs, filtering for DRIGs, further selecting genes associated with GC prognosis, constructing a predictive model and validating it using external datasets ACRG and PUMCH (Fig. 1). First, the four DRGs were identified and obtained from the previous literature [18, 30]. The expression of *SLC7A11*, *SLC3A2*, *RPN1* and *NCKAP1* were found to be upregulated in the tumor tissues compared with that in the corresponding normal tissues from the TCGA data (Fig. 2A). The mutation profile of these four DRGs was detected in the GC samples. Among the four genes, *SLC7A11*, *RPN1* and *NCKAP1* exhibited minimal alterations, whereas *SLC3A2* showed a higher mutation rate in the 431 GC samples (Fig. 2B). Further correlation analysis of expression levels in GC samples confirmed the synergistic effects of the four DRGs in disulfidptosis (Fig. 2C). Additionally, the correlation between the DRGs and immune cells was examined. *NCKAP1* showed predominantly positive correlations with M2 macrophages, while displaying negative correlations to regulatory T cells. *RPN1*, *SLC3A2* and *SLC7A11* were found to positively activate mast cells, whereas *RPN1* and *SLC3A2* were negative correlations to memory B cells. And *SLC7A11* was negative correlations to regulatory T cells (Fig. 2D).

To validate the expression of *SLC7A11* in GC tissues, a tissue array containing 63 paired samples was utilized. *SLC7A11*, known to play a role in the ferroptosis process and chemoresistance in various tumors [31, 32]. As shown in Fig. 2E, the IOD of IHC revealed that *SLC7A11* was significantly increased in the GC tissues. The representative images of IHC showed that *SLC7A11* was elevated in the GC tissues and mainly located in the cytoplasm (Fig. 2F). The correlation between *SLC7A11* expression and clinicopathological characteristics revealed that higher expression of *SLC7A11* was found to be associated with worse T stage, N stage, and AJCC stage in GC patients from the PUMCH cohort (Table 1).

**Table 1:** Relationship between clinicopathological characteristics and expression of VPS35, HIF-1α, GLA, CDC37 and SLC7A11 in gastric cancer (*n* = 63)

	Expression of VPS35		Expression of HIF-1α		Expression of GLA		Expression of CDC37		Expression of SLC7A11	
	High (*n* = 32)	Low (*n* = 31)	High (*n* = 32)	Low (*n* = 31)	High (*n* = 32)	Low (*n* = 31)	High (*n* = 32)	Low (*n* = 31)	High (*n* = 32)	Low (*n* = 31)
Gender										
Male	19	23	20	22	22	20	22	20	19	23
Female	13	8	12	9	10	11	10	11	13	8
Age (years)										
< 55	5	8	6	7	4	9	6	7	4	9
≥ 55	27	23	26	24	28	22	26	24	28	22
Tumor location										
Fundus	7	6	6	7	8	5	7	6	8	5
Body	12	15	16	11	11	16	15	12	15	12
Antrum	13	10	10	13	13	10	10	13	9	14
Tumor size (cm)										
≤ 3	**17**	**25**	**17**	**25**	18	24	19	23	18	24
> 3	**15**	6*	**15**	6*	14	7	13	8	14	7
Tumor T stage										
1 + 2	20	24	20	24	**16**	**28**	21	23	**18**	**26**
3 + 4	12	7	12	7	**16**	3***	11	8	**14**	5*
Tumor N stage										
0	15	19	**13**	**21**	**13**	**21**	**10**	**24**	**13**	**21**
1 + 2 + 3	17	12	**19**	10*	**19**	10*	**22**	7***	**19**	10*
AJCC stage										
I + II	**21**	**27**	**20**	**28**	**21**	**27**	**20**	**28**	**21**	**27**
III	**11**	4*	**12**	3**	**11**	4*	**12**	3**	**11**	4*
Differentiation										
Well-moderately	11	8	8	11	10	9	11	8	9	10
Poorly	21	23	24	20	22	22	21	23	23	21

Statistical significance was determined by the chi-square test. (*p < 0.05, **p < 0.01)

### The distribution and expression of four DRGs in GC at single-cell level

To explore the DRGs in GC at the single-cell level, scRNA-seq data was obtained from the GSE183904 dataset. First, the data was screened with quality control through detected gene numbers, the depth of sequencing, and the gene ratio of mitochondrial and hemoglobin in each specimen (Figures S1A and S1B). Following data normalization, 3000 variable genes were chosen to further analysis (Figure S1C). PCA analysis was used to reduce dimensionality and visualize the data, and the top 30 principal components (PCs) were selected as the input for UMAP analysis (Figure S1D).

**Fig. 3:**
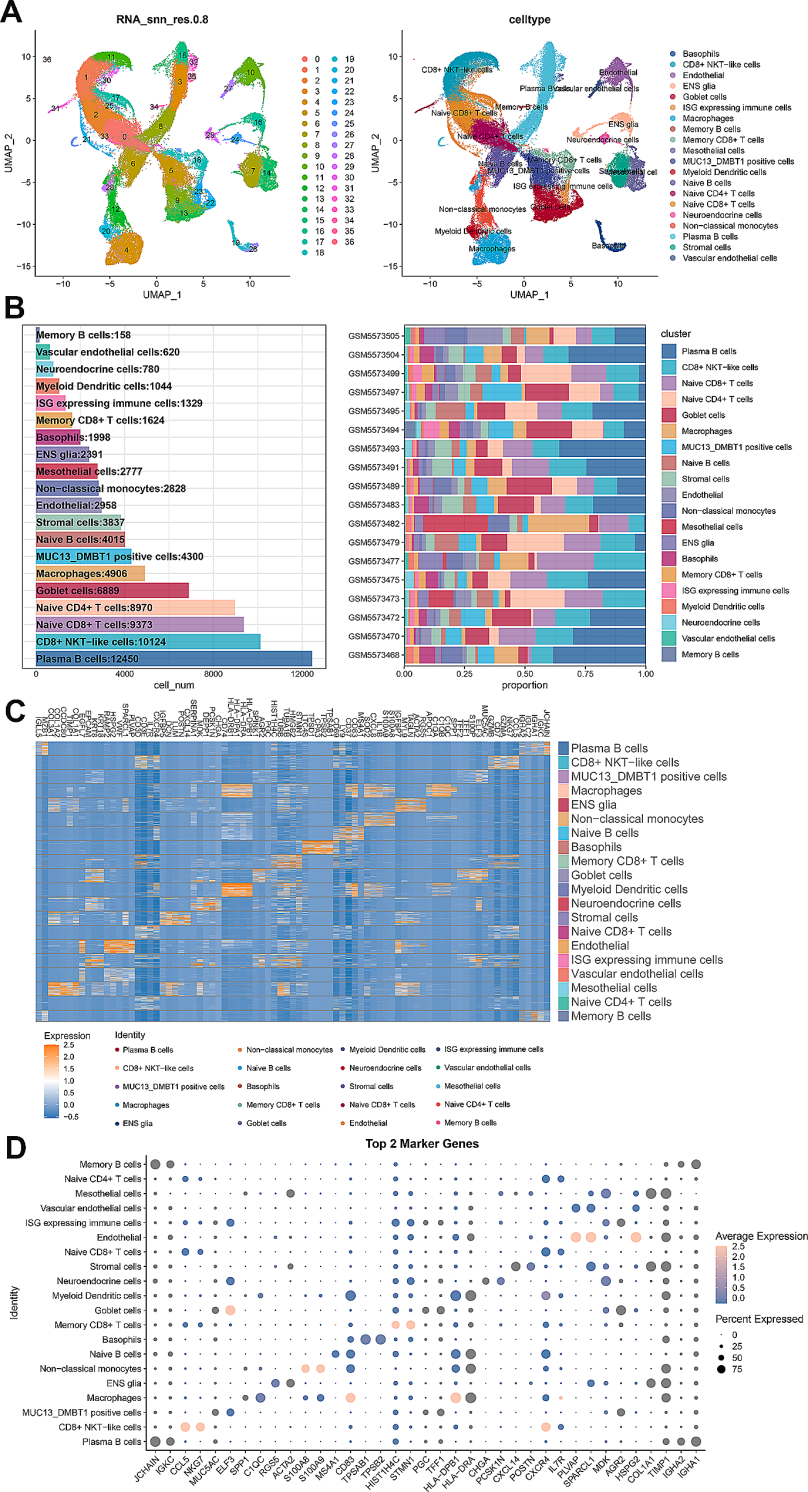
The analysis of single-cell RNA sequencing. **A** UMAP of 37 independent cell clusters and 20 cell types were identified by marker genes. **B** The total number of each cell type and distribution of cell types in different specimens. **C** The heatmap shows the top 5 marker genes in each annotated cell type. **D** The bubble diagram shows the top 2 marker genes in each annotated cell type

Subsequently, UMAP dimensionality reduction resulted in the classification of cells into 37 clusters (Fig. 3A). These 37 clusters were classified into 20 cell types by using SingleR (basophils, CD8^+^ NKT − like cells, endothelial, ENS glia, goblet cells, ISG expressing immune cells, macrophages, memory B cells, memory CD8^+^ T cells, mesothelial cells, MUC13_DMBT1 positive cells, myeloid dendritic cells, naive B cells, naive CD4^+^ T cells, naive CD8^+^ T cells, neuroendocrine cells, non − classical monocytes, plasma B cells, stromal cells and vascular endothelial cells) (Fig. 3A). Additionally, the specific number and the proportion of each cell type in each GC specimen were calculated (Fig. 3B). The heatmap presented the top 5 marker genes of each cell type (Fig. 3C), while the bubble diagram presented the top 2 marker genes in each cell type (Fig. 3D). The UMAP distribution presented the most representative marker gene for each cell type (Figure S2).

**Fig. 4:**
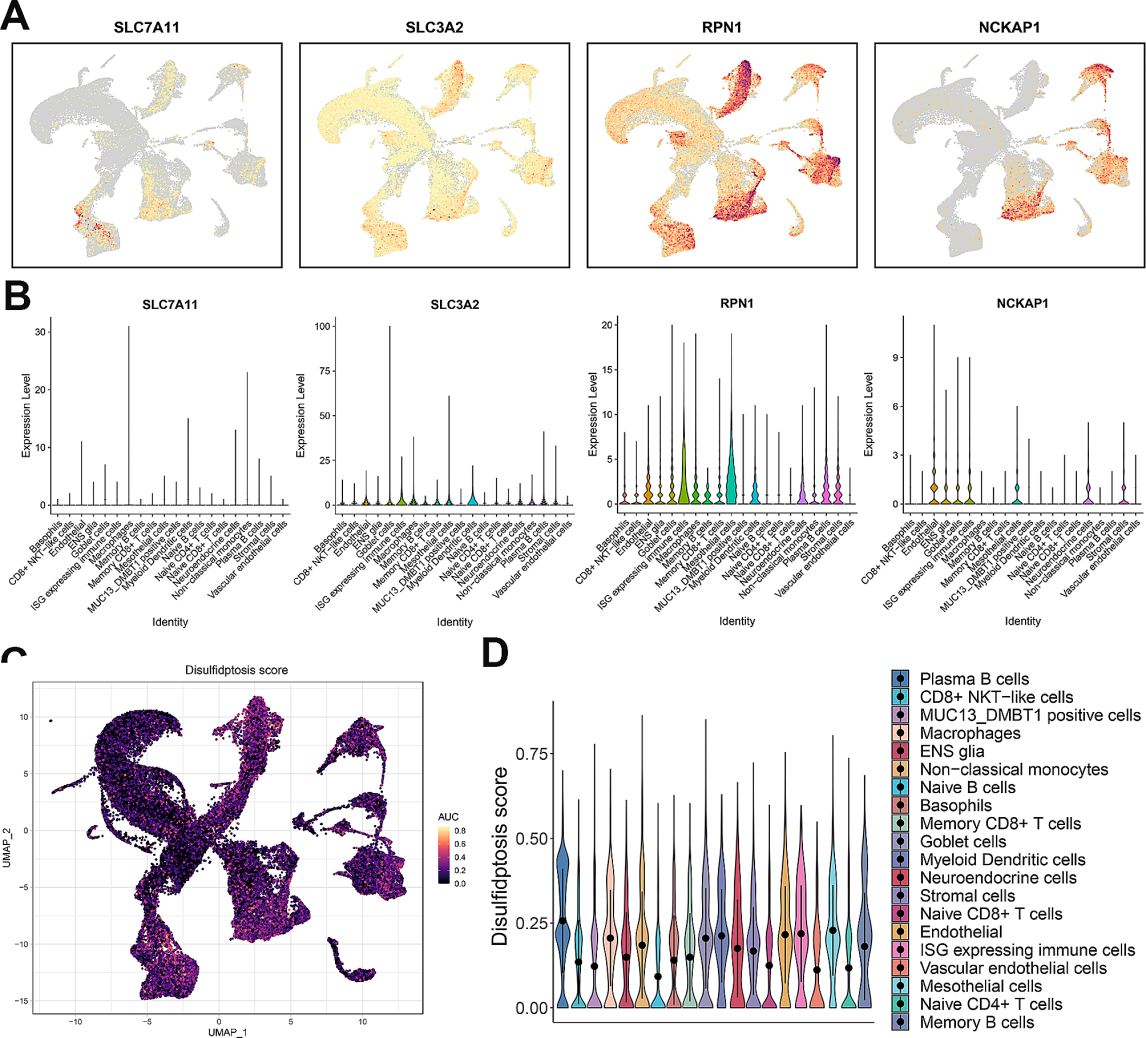
The expression of disulfidptosis-related genes in GC at single cell level. **A** The UMAP distribution of SLC7A11, SLC3A2, RPN1 and NCKAP1 abundance in GC at single cell level. The color variation represents the relative level of gene expression within different cellular clusters, with red and yellow areas indicating higher gene expression, and grey areas indicating lower gene expression or non-detection. **B** Violin plots of SLC7A11, SLC3A2, RPN1 and NCKAP1 abundance in GC at single cell level. **C** UMAP distribution of disulfidptosis score of cells by using AUCell function. **D** Violin plots of disulfidptosis score of each cell type

Examining the single-cell expression profiles of *SLC7A11*, *SLC3A2*, *RPN1* and *NCKAP1* in GC, as depicted in UMAP distribution (Fig. 4A) and violin plots (Fig. 4B), revealed a prevalent disulfidptosis signature. Each cell’s disulfidptosis score, derived from the expression of the four DRGs using the ‘AUCell’ function, was effectively illustrated in the UMAP distribution (Fig. 4C) and visually represented for each cell type through violin plots (Fig. 4D). Of particular note is the heightened disulfidptosis score observed in plasma B cells at the single-cell level. This observation suggests the potential significance of plasma B cells within the gastric cancer tumor microenvironment. These results contribute valuable insights into the distribution and relevance of disulfidptosis across diverse cell types, enriching our understanding of its role in GC.

### Screening of differentially expressed DRIGs in GC

**Fig. 5:**
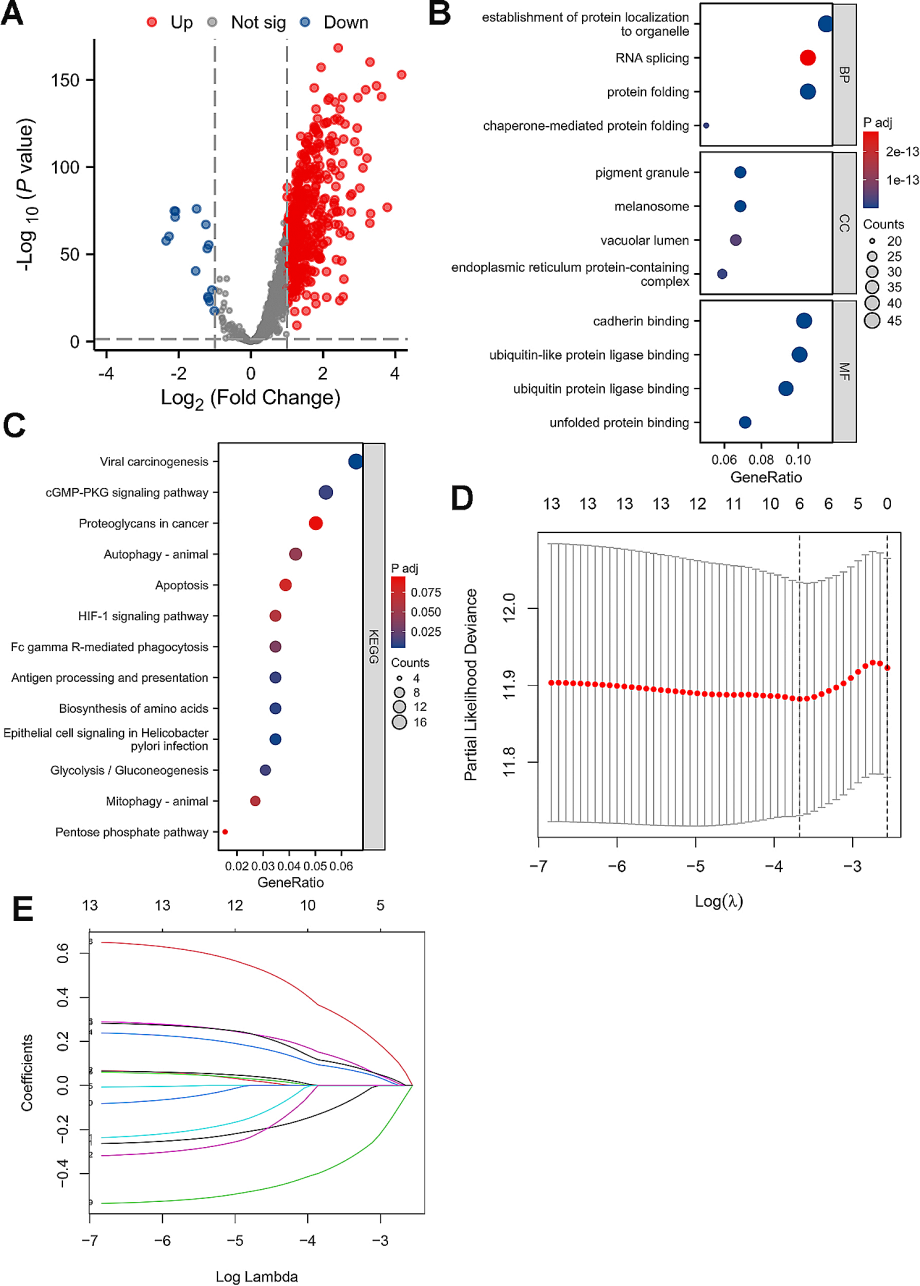
The screen of disulfidptosis-related immune genes. **A** The volcanic plot of differentially expressed disulfidptosis-related immune genes in GC. **B** The GO analysis of differentially expressed disulfidptosis-related immune genes in GC. **C** The KEGG analysis of differentially expressed disulfidptosis-related immune genes in GC. **D** LASSO Cox regression analysis of the association between deviance and log(λ). **E** LASSO Cox regression analysis of the association between coefficients of genes and log(λ)

To reveal the intrinsic correlations between IRGs and DRGs, a total of 474 differentially expressed DRIGs (459 upregulated DRIGs and 15 downregulated DRIGs) were screened out in GC via the criteria of *p* < 0.05 and Log_2_ (fold change) > 1 (Fig. 5A). These DRIGs exhibited enrichment in the establishment of protein localization to organelle in biological process, pigment granule in cellular component, cadherin binding in molecular function, viral carcinogenesis and cGMP-PKG signaling pathway to aggravate the progression of GC through using GO and KEGG analysis (Fig. 5B and C; Table 2). Then, univariate Cox and LASSO analyses were utilized to filter the prominent DRIGs from 474 differentially expressed DRIGs for the prognosis of GC patients (Fig. 5D and E). *GLA*, *HIF-1α*, *VPS35* and *CDC37* were successfully identified from 474 differentially expressed DRIGs to forecast the survival time of GC patients by using the abovementioned methods.

**Table 2:** The GO and KEGG enrichment analysis of disulfidptosis-related immune genes

Ontology	ID	Description	GeneRatio	BgRatio	p value	p.adjust
BP	GO:0006457	protein folding	42/399	212/18,800	1.13e-28	4.49e-25
BP	GO:0072594	establishment of protein localization to organelle	46/399	431/18,800	1.3e-19	2.6e-16
BP	GO:0061077	chaperone-mediated protein folding	20/399	70/18,800	1.31e-17	1.75e-14
CC	GO:0042470	melanosome	28/408	109/19,594	5.77e-23	1.48e-20
CC	GO:0048770	pigment granule	28/408	109/19,594	5.77e-23	1.48e-20
CC	GO:0140534	endoplasmic reticulum protein-containing complex	24/408	125/19,594	1.06e-16	1.8e-14
MF	GO:0051082	unfolded protein binding	29/407	121/18,410	4.18e-22	2.58e-19
MF	GO:0045296	cadherin binding	42/407	333/18,410	4.67e-20	1.07e-17
MF	GO:0044389	ubiquitin-like protein ligase binding	41/407	317/18,410	5.21e-20	1.07e-17
KEGG	hsa05203	Viral carcinogenesis	17/259	204/8164	0.0003	0.0046
KEGG	hsa05120	Epithelial cell signaling in Helicobacter pylori infection	9/259	70/8164	0.0003	0.0055
KEGG	hsa01230	Biosynthesis of amino acids	9/259	75/8164	0.0006	0.0087
KEGG	hsa04612	Antigen processing and presentation	9/259	78/8164	0.0008	0.0110
KEGG	hsa04022	cGMP-PKG signaling pathway	14/259	167/8164	0.0008	0.0114
KEGG	hsa00010	Glycolysis / Gluconeogenesis	8/259	67/8164	0.0012	0.0136
KEGG	hsa04666	Fc gamma R-mediated phagocytosis	9/259	97/8164	0.0035	0.0347
KEGG	hsa04140	Autophagy - animal	11/259	141/8164	0.0051	0.0452
KEGG	hsa04137	Mitophagy - animal	7/259	72/8164	0.0076	0.0614
KEGG	hsa04066	HIF-1 signaling pathway	9/259	109/8164	0.0076	0.0614
KEGG	hsa04210	Apoptosis	10/259	136/8164	0.0111	0.0780
KEGG	hsa05205	Proteoglycans in cancer	13/259	205/8164	0.0134	0.0918
KEGG	hsa00030	Pentose phosphate pathway	4/259	30/8164	0.0142	0.0947

### Construction and validation of the DRIG signature

The formulation of the risk score formula and calculation of gene coefficients were performed using multivariate Cox analysis. The formula to score GC patients was obtained as follows: Risk score = (-0.25455 × *GLA*) + (0.29278 × *HIF-1α*) + (0.62703 × *VPS35*) + (-0.52510 × *CDC37*). Then, patients were classified into high-risk and low-risk groups based on the median value of all patients’ risk scores.

**Fig. 6:**
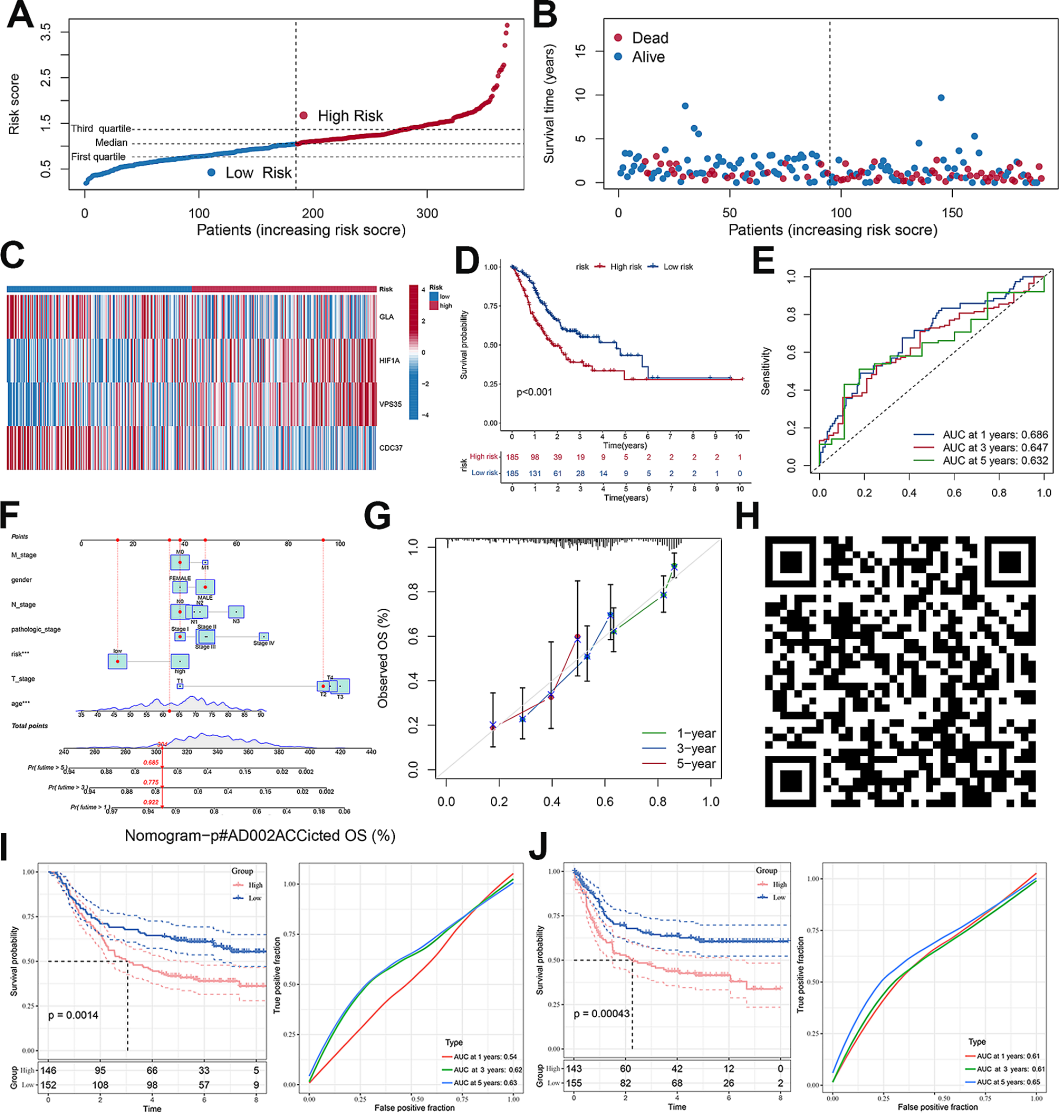
Construction and validation of prognostic models based on disulfidptosis-related immune genes. **A** Distribution of risk score. **B** The survival status and survival time of GC patients ranked by risk score. **C** The heatmap of GLA, HIF-1 A, VPS35 and CDC37 in two groups. **D** Kaplan-Meier analysis between high-risk group and low-risk group. **E** Time-dependent ROC curve of risk score predicting the 1-, 3-, and 5-year overall survival. **F** Details of the nomogram. **G** The calibration curve for predicting 1-, 3-, and 5-year overall survival. **H** The quick response code of online dynamic nomogram **I** The OS discrepancy between the high-risk and low-risk groups and the ROC curve of risk score predicting the 1-, 3-, and 5-year OS using the GSE62254 dataset. **J** The DFS discrepancy between the high-risk and low-risk groups and the ROC curve of risk score predicting the 1-, 3-, and 5-year DFS using the GSE62254 dataset

As shown in Fig. 6A and B, the proportion of death was higher in the GC patients with high risk scores. The heatmap displayed the expression of four DRIGs in two groups, in which *HIF-1α* and *VPS35* were increased in the high-risk group while *GLA* and *CDC37* were elevated in the low-risk group (Fig. 6C). In addition, KM analysis showed that patients with high scores had shorter survival times compared with patients with low scores (Fig. 6D). The risk signature was then utilized to predict the survival probability of GC patients. The accuracy of signature in predicting the 1-, 3-, and 5-year probability was 0.686, 0.647 and 0.632 in GC patients, respectively (Fig. 6E). A nomogram consisting of a risk model and various clinicopathological features was successfully established to accurately forecast the survival time of GC patients (Fig. 6F). The calibration curve was used to verify the validity and accuracy of the nomogram for the predictive probability of 1-, 3- and 5-year in GC patients (Fig. 6G). Further, a webserver (https://pumc.shinyapps.io/GastricCancer/) was constructed to make full use of the nomogram in prognostic prediction for GC patients. The quick response code provided a convenient entrance using the online tool in clinical practice for physicians (Fig. 6H). For the stringency of the study, the Asian Cancer Research Group cohort (GSE62254) was utilized to validate the clinical values of the signature. The signature not only well distinguished the overall survival (OS) and disease-free survival (DFS) between the two groups, but also accurately predicted the 1-, 3- and 5-year OS and DFS of GC patients (Fig. 6I and J). In summary, we preliminarily investigated and validated the potential use of DRIGs in predicting prognosis of GC patients in clinical translation.

### Gene Set Enrichment Analysis (GSEA) and immune cell infiltration in two groups

**Fig. 7:**
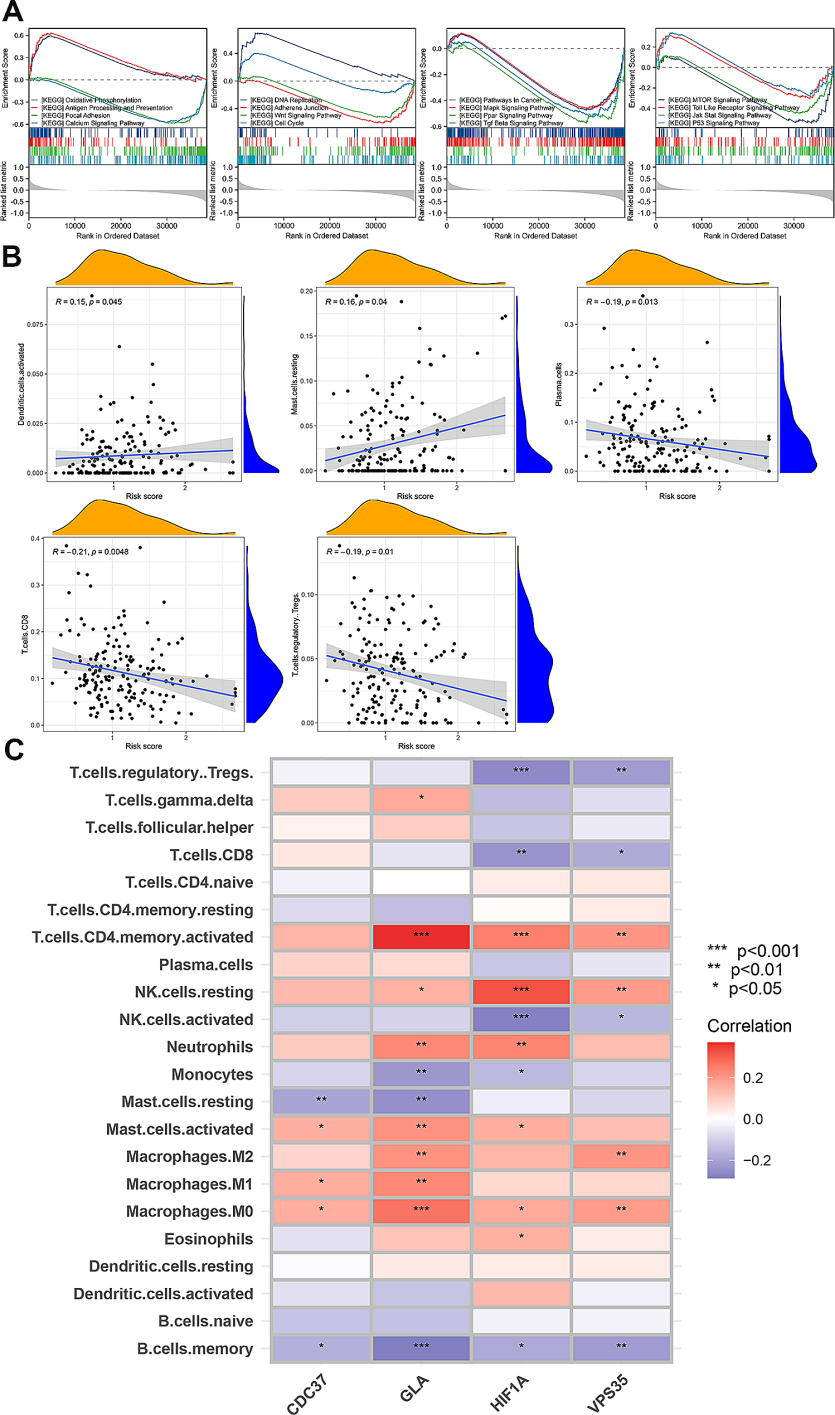
The GSEA results and correlation between the risk signature and immune cell infiltrations. **A** The results of GSEA analysis between high-risk group and low-risk group. **B** The correlation between the risk score and infiltrated immune cells. **C** The correlation between the GLA, HIF-1 A, VPS35 and CDC37 and infiltrated immune cells

The discrepancy in survival time between high-risk and the low-risk groups suggests a significant heterogeneity of the genome in the two groups. To further investigate the underlying mechanisms contributing to this heterogeneity, GSEA analysis was used to further explore the gaps in underlying mechanisms between the two groups. As shown in Fig. 7A, multiple classical signaling pathways of tumors were involved in the high-risk group, including the activation of oxidative phosphorylation, antigen processing and presentation, DNA replication and cell cycle and inhibition of focal adhesion, calcium signaling pathway, adherens junction, Wnt signaling pathway, pathways in cancer, MAPK pathway, PPAR pathway, TGF beta pathway, mTOR pathway, Toll-like receptor pathway, JAK-STAT pathway and P53 pathway. The involvement of these signaling pathways resulted in the exacerbation of GC progression. In addition, the correlation between risk score and immune cell infiltration was also evaluated. The result revealed that risk score was positively correlated to the activation of dendritic cells and resting of mast cells, while negatively related to the plasma cells, CD8^+^ T cells and regulatory T cells (Fig. 7B). Furthermore, the correlation between 4 DRIGs and immune cell infiltration was also investigated. As shown in Fig. 7C, *CDC37* was positively correlated to the activation of mast cells, M0 and M1 macrophages. *GLA* was positively correlated to the activation of memory CD4^+^ T cells while negatively related to memory B cells, *HIF-1α* was positively related to the activation of memory CD4^+^ T cells while negatively correlated to the activation of NK cells, *VPS35* was negatively related to regulatory T cells while positively correlated to the activation of memory CD4^+^ T cells and M2 macrophages. The data revealed that the 4 DIRGs (*CDC37*, *GLA*, *HIF-1α* and *VPS35*) that constructed the risk signature profoundly regulated the immune microenvironment of GC.

### Somatic mutation profile of 4 DIRGs and two groups

**Fig. 8:**
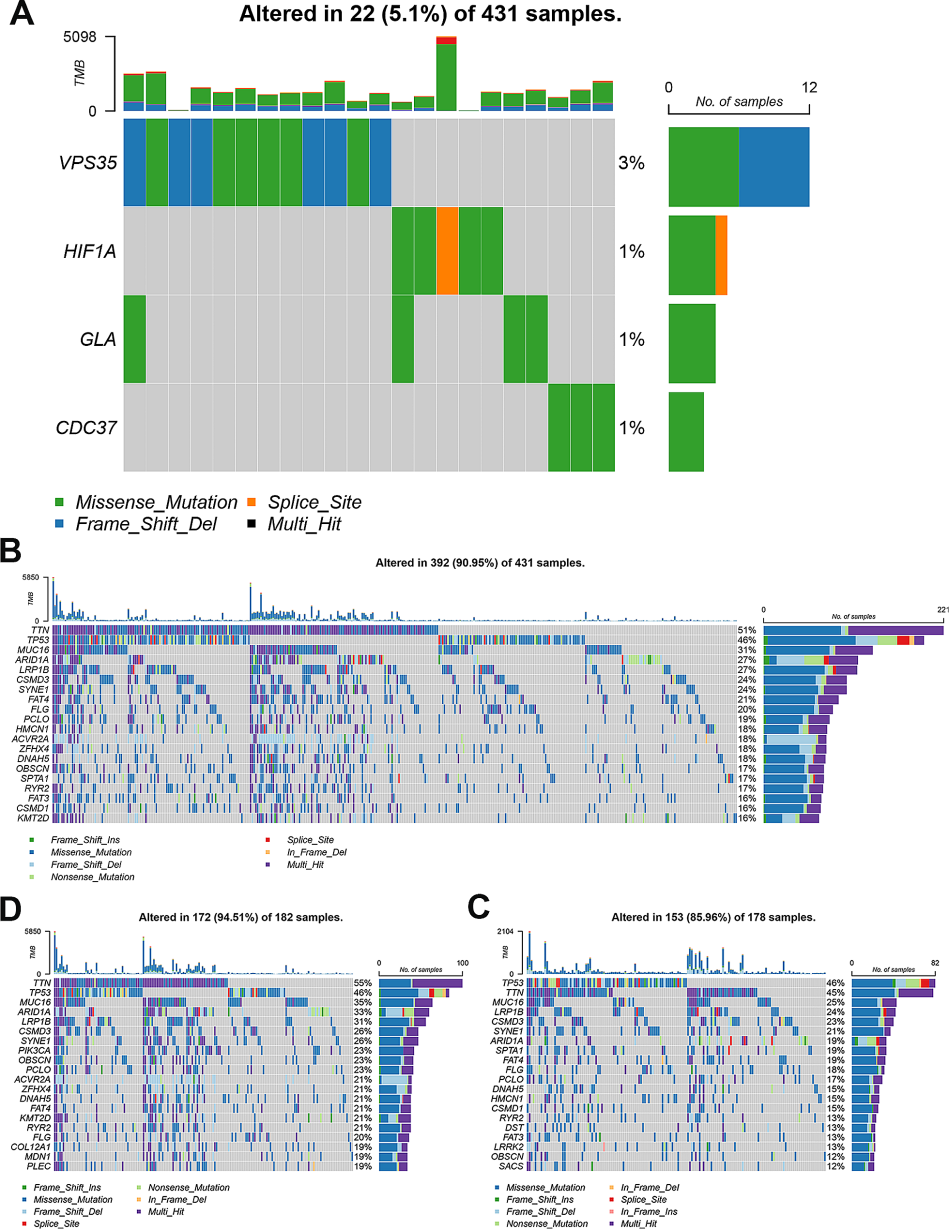
Somatic mutations in the entire GC samples and two groups. **A** Mutation profile of GLA, HIF-1 A, VPS35 and CDC37 in 431 GC patients. **B** The mutation profile in low-risk group. **C** The mutation profile in high-risk group. **D** The mutation profile in entire GC samples

First, the mutation of *CDC37*, *GLA*, *HIF-1α* and *VPS35* was investigated in the 431 GC specimens. The results indicated that 4 DIRGs were generally stable in GC and only 5.1% of samples exhibiting mutations in *CDC37*, *GLA*, *HIF-1α* and *VPS35* (Fig. 8A). The results of somatic mutation in 431 GC specimens revealed that *TTN*, *TP53*, *MUC16*, *ARID1A*, *LRP1B*, *CSMD3*, *SYNE1*, *FAT4*, *FLG* and *PCLO* were among the top 10 genes with the highest mutation frequencies (Fig. 8B). Then, the mutation profile in the two groups was also detected. *TTN*, *TP53*, *MUC16*, *ARID1A*, *LRP1B*, *CSMD3* and *SYNE1* were the most frequently mutated genes in both groups. However, *PIK3CA*, *OBSCN* and *PCLO* were the three most frequently mutated genes in the low-risk group (Fig. 8C), while *SPTA1*, *FAT4* and *FLG* were the frequently mutated genes in the high-risk group (Fig. 8D).

### The clinical application of risk signature in GC

**Fig. 9:**
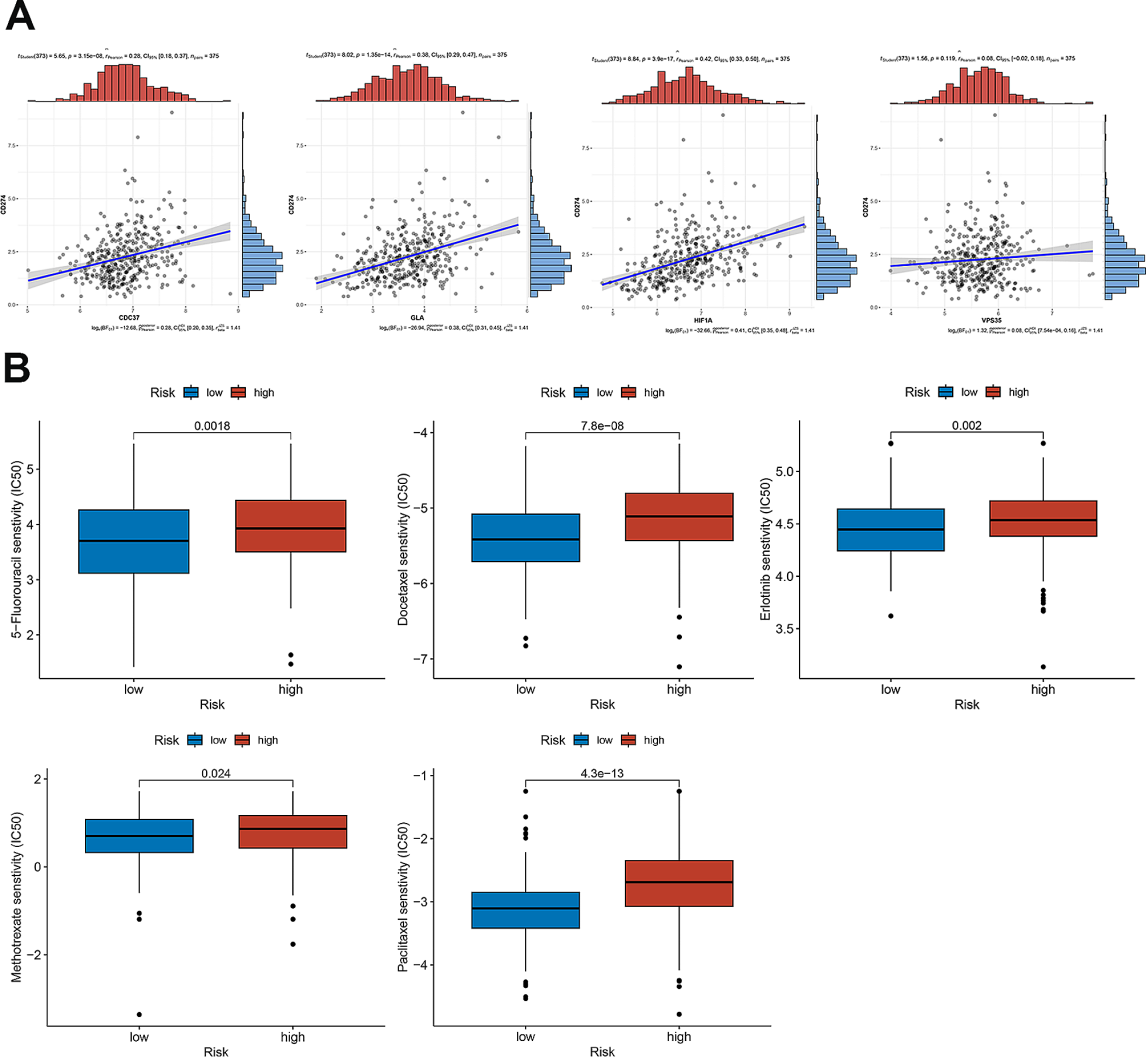
Immune infiltration level and drug sensitivity analysis based on the risk model. **A** The correlation between the GLA, HIF-1 A, VPS35 and CDC37 and CD274. **B** The results of drug sensitivity analysis between high-risk and low-risk groups

A cruel situation is that about 80% of hospitalized patients were initially diagnosed with locally advanced or metastatic GC in China [8, 33]. This phenomenon required postoperative chemotherapy and immunotherapy in treatment for GC patients. Therefore, we explored the relationship between 4 DIRGs and *CD274* expression, as well as the response to classical chemotherapy drugs of GC in the two groups. As shown in Fig. 9A, the expression of *CD274* was usually consistently elevated in parallel with the increased expression of *CDC37*, *GLA*, *HIF-1α* and *VPS35* in GC. This phenomenon indirectly reflected that 4 DRIGs may be associated to the immune evasion of GC cells. In addition, patients with higher risk scores were generally more resistant to 5 − Fluorouracil, docetaxel, erlotinib, methotrexate and paclitaxel treatments in GC (Fig. 9B). These findings highlight a potential risk signature in guiding therapy selection for GC patients in the future.

### Validation of 4 DIRGs in GC using PUMCH cohort

**Fig. 10:**
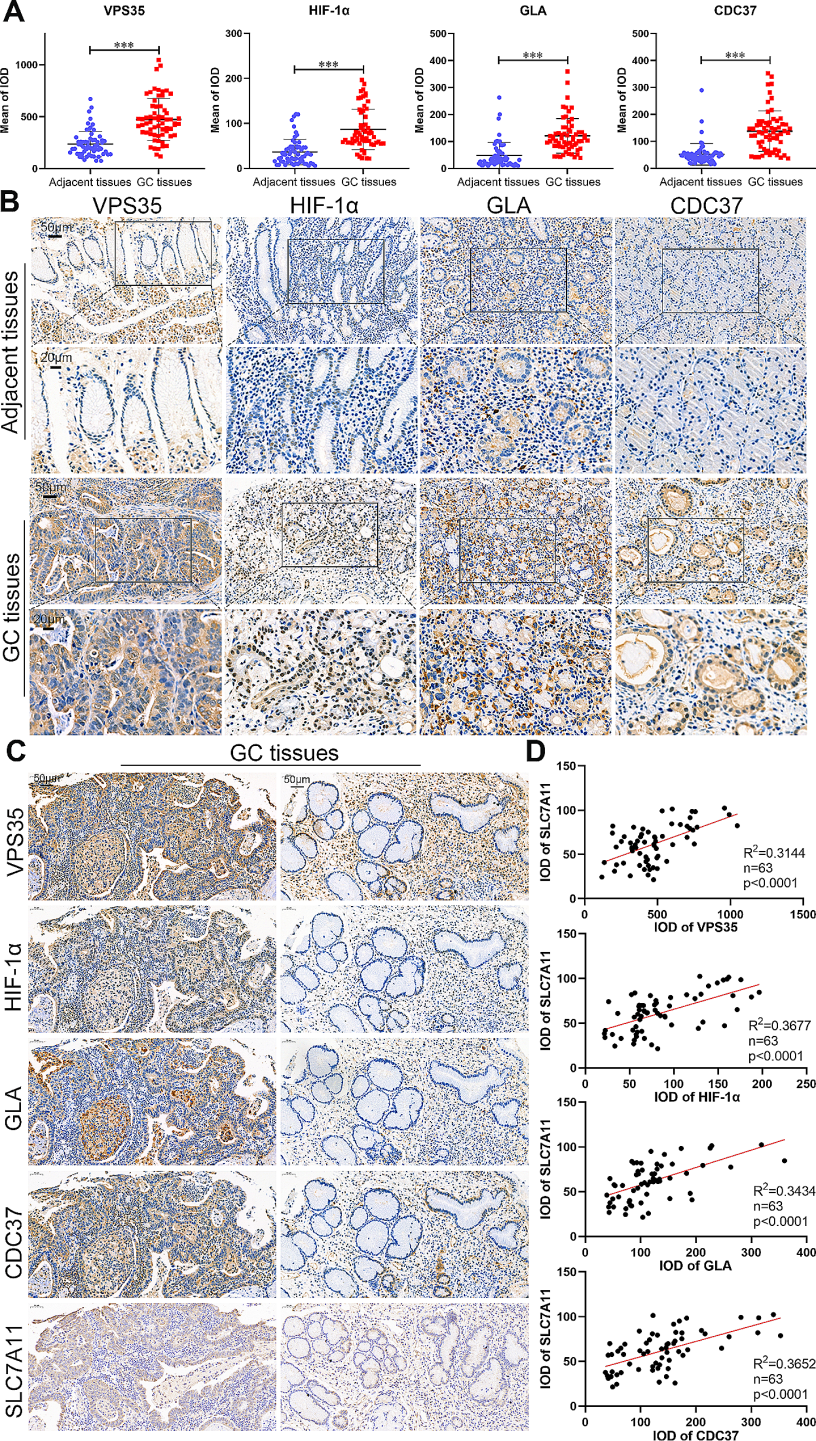
The expression of GLA, HIF-1 A, VPS35, CDC37 and SLC7A11 in GC. **A** The expression of GLA, HIF-1 A, VPS35 and CDC37 in 63 paired GC and adjacent normal tissues. **B** The IHC of GLA, HIF-1 A, VPS35 and CDC37 in GC and adjacent normal tissues (scale bar: 50 μm and 20 μm). **C** The representative IHC images of GLA, HIF-1 A, VPS35, CDC37 and SLC7A11 in the same GC tissues (scale bar: 50 μm). **D** The correlation between GLA, HIF-1 A, VPS35, CDC37 and SLC7A11 in the GC tissues. Student’s t-test was used to determine statistical significance: ***p < 0.001

The PUMCH cohort were then used to validate the expression of *CDC37*, *GLA*, *HIF-1α* and *VPS35*, as well as their relationships to *SLC7A11* and clinicopathological characteristics in GC for the abovementioned multiple explorations in bioinformatics results. The expression of *CDC37*, *GLA*, *HIF-1α* and *VPS35* was detected in the tissue array by IHC staining. The results of IHC demonstrated that the expression of *CDC37*, *GLA*, *HIF-1α* and *VPS35* was upregulated in the tumor tissues compared with corresponding normal tissues (Fig. 10A and B). Additionally, separate IHC staining of the same GC tissue demonstrated that the expression of *SLC7A11* increased with elevated level of *CDC37*, *GLA*, *HIF-1α* or *VPS35* (Fig. 10C). Analysis of data from 63 GC tissues showed a positive correlation between *CDC37*, *GLA*, *HIF-1α*, *VPS35* and *SLC7A11* (Fig. 10D). Furthermore, patients with higher expressions of *VPS35* and *HIF-1α* frequently associated with larger tumor size; while higher expression of *GLA* was indicative of worse T stage, N stage and AJCC stage. Increased expressions of *CDC37* and *HIF-1α* often predicted worse N stage and advanced clinical stage of GC (Table 1). The close relationships between the 4 DIRGs and clinicopathological characteristics further support the validity and accuracy of the disulfidptosis-related immune genes signature in predicting the survival time of GC patients.

## Discussion

Due to the environment, diet and *Helicobacter pylori* infection, China has always been a region with a high incidence of GC, as well as the low penetration rate in early screening of GC, about 80% of patients are in the advanced stage at the time of diagnosis, which also leads to a high mortality of GC in China [8, 34]. This phenomenon warranted subgroup classification of patients and selection of postoperative adjuvant chemotherapy and immune checkpoint inhibitors for GC treatment. Therefore, establishing a signature for risk stratification and long-term survival prediction of patients will be of great benefit to improve the overall survival of patients with GC.

The function and activation of infiltrated immune cells in TME are influenced by various factors, such as signal molecules or metabolites [35–37]. Disulfidptosis, a newly discovered type of cell death caused by disulfide stress, may be highly related to immune response in tumors [38, 39]. Several studies have reported the relationship between DRGs and immune cell infiltrations in TME and the prognostic value of DRGs for patients, including hepatocellular carcinoma [40, 41], renal cell carcinoma [42], bladder cancer [43] and lung adenocarcinoma [38, 44]. However, the intrinsic relationship and regulatory network between cell disulfidptosis and immune cell activities and the prognostic value of DRGs for patients are currently blank in GC. Hence, it is necessary to explore the intrinsic mechanism of cell disulfidptosis and immune functions and use DRIGs to build a robust prognostic signature in GC.

The four key DRGs (*SLC7A11*, *SLC3A2*, *RPN1* and *NCKAP1*) were found to be upregulated and highly conserved in GC from analyzing the TCGA data. Furthermore, the close relationship between DRGs and the activities of various immune cells indicated the existence of a potential regulatory network. scRNA-seq can accurately evaluate the role of genes in specific cell types after cell clustering, which facilitates the exploration of molecular mechanisms in tumorigenesis and development [45, 46]. Hence, the distribution and expression of DRGs were investigated in GC at the single-cell level after clustering the cells into 20 cell types. The results revealed that these DRGs not only indirectly affected the immune cells function by regulating the disulfidptosis of tumor cells, but also potentially directly influenced immune cell activities. Then, we performed Pearson correlation analysis to screened out the differentially expressed IRGs closely to the cell disulfidptosis in GC. Functional enrichment analysis demonstrated that DRIGs were involved in the regulation of various biological processes and key signaling pathways and transduction. Four DRIGs (*CDC37*, *GLA*, *HIF-1α* and *VPS35*) were chosen from 474 differentially expressed DRIGs to form a risk score formula by LASSO and COX analysis. The formula was then applied to 360 GC patients with integrated clinical information and divided into two groups based on the mean value. Survival analysis validated that the model can effectively discriminate the long-term survival of patients between the two groups. In addition, when the signature was combined with TNM staging, the developed nomogram overcame the limitations of TNM staging alone and greatly improved the accuracy of prognostic prediction for GC patients. Further, the risk signature also worked in the prognostic prediction and effectively distinguished the survival discrepancy of GC patients in the ACRG cohort.

GESA analysis was used to excavate the underlying mechanisms between the two groups. The discrepancy in the activation and inhibition of signal pathways explained the discrepancy in the survival time between the two groups at the molecular mechanism level. In addition, high risk scores often indicated a favorable immune microenvironment (IME) for tumor progression whereas low risk scores meant an unfavorable IME for tumor progression. The variation in mutation profiles between the two groups can guide treatment methods and drug selection for patients with GC. As an abovementioned phenomenon, advanced-stage GC patients accounted for a high proportion in China, making postoperative chemotherapy essential for these advanced-stage GC patients [9]. In recent years, the emergence of ICIs, such as *CD274*, has brought the dawn of treatment for patients, and it was the first-line treatment for advanced-stage GC patients [47, 48]. The signature can be used to guide the selection of sensitive chemotherapeutic drugs and ICIs for GC patients, thereby improving the survival time of patients. For the rigor and accuracy of the study, we detected the expression of *GLA*, *HIF-1α*, *VPS35*, *CDC37* and *SLC7A11* by IHC in 63 paired GC tissues and adjacent normal tissues. Consistent with the transcriptome expression in the TCGA database, the protein expression of *GLA*, *HIF-1α*, *VPS35*, *CDC37* and *SLC7A11* was also significantly increased in GC tissues. Furthermore, the expression of *GLA*, *HIF-1α*, *VPS35*, *CDC37* and *SLC7A11* was closely associated with the clinicopathological characteristics of GC patients. Moreover, the positive correlations among *GLA*, *HIF-1α*, *VPS35*, *CDC37* and *SLC7A11* in the GC tissues were also validated. Next, in vivo and in vitro experiments and exploration of molecular mechanisms between immune infiltration and cell disulfidptosis in GC need to be gradually implemented.

## Conclusions

In summary, our study successfully elucidated the potential of DRIGs as biomarkers in GC and developed a signature consisting of four DRIGs that effectively predicts patient prognosis and clinicopathological characteristics. The findings of this study have significant implications for guiding the understanding of immune infiltration and cell disulfidptosis in GC and informing the selection of chemotherapy drugs and ICIs for future patient treatment strategies.

### Electronic supplementary material

Below is the link to the electronic supplementary material.

Supplementary Material 1

Supplementary Material 2

Supplementary Material 3

## Data Availability

The original data and the generated analysis data in the research process can be obtained from the corresponding authors under reasonable requirements.

## Abbreviations

GC: Gastric Cancer
IRGs: Immune-Related Genes
scRNA-seq: single-cell RNA Sequencing
DRGs: Disulfidptosis-Related Genes
TNM: Tumor-Node-Metastasis
TME: Tumor Microenvironment
PUMCH: Peking Union Medical College Hospital
UMAP: Uniform Manifold Approximation and Projection
IHC: Immunohistochemistry
IOD: Integrated Optical Density
ICIs: Immune Checkpoint Inhibitors
IME: Immune Microenvironment

## References

[1] Parkin DM, Pisani P, Ferlay J (1999). Global cancer statistics. Cancer J Clin.

[2] Parkin DM, Bray F, Ferlay J, Pisani P (2005). Global cancer statistics, 2002. Cancer J Clin.

[3] Jemal A, Bray F, Center MM, Ferlay J, Ward E, Forman D (2011). Global cancer statistics. Cancer J Clin.

[4] Torre LA, Bray F, Siegel RL, Ferlay J, Lortet-Tieulent J, Jemal A (2015). Global cancer statistics, 2012. Cancer J Clin.

[5] Bray F, Ferlay J, Soerjomataram I, Siegel RL, Torre LA, Jemal A (2018). Global cancer statistics 2018: GLOBOCAN estimates of incidence and mortality worldwide for 36 cancers in 185 countries. Cancer J Clin.

[6] Sung H, Ferlay J, Siegel RL, Laversanne M, Soerjomataram I, Jemal A, Bray F (2021). Global Cancer statistics 2020: GLOBOCAN estimates of incidence and Mortality Worldwide for 36 cancers in 185 countries. CA Cancer J Clin.

[7] Wong MCS, Huang J, Chan PSF, Choi P, Lao XQ, Chan SM, Teoh A, Liang P (2021). Global incidence and mortality of gastric Cancer, 1980–2018. JAMA Netw open.

[8] Zheng R, Zhang S, Zeng H, Wang S, Sun K, Chen R, Li L, Wei W, He J. Cancer incidence and mortality in China, 2016. J Natl Cancer Cent 2022. 10.1016/j.jncc.2022.02.002 PMC11256658 39035212

[9] Johnston FM, Beckman M (2019). Updates on management of gastric Cancer. Curr Oncol Rep.

[10] Park KB, Jun KH, Song KY, Chin H, Lee HH (2022). Development of a staging system and survival prediction model for advanced gastric cancer patients without adjuvant treatment after curative gastrectomy: a retrospective multicenter cohort study. Int J Surg (London England).

[11] Li J, Yu T, Sun J, Zeng Z, Liu Z, Ma M, Zheng Z, He Y, Kang W (2023). Comprehensive analysis of cuproptosis-related immune biomarker signature to enhance prognostic accuracy in gastric cancer. Aging.

[12] Zhao Z, Mak TK, Shi Y, Huang H, Huo M, Zhang C (2023). The DNA damage repair-related lncRNAs signature predicts the prognosis and immunotherapy response in gastric cancer. Front Immunol.

[13] Liu Y, Zheng H, Gu AM, Li Y, Wang T, Li C, Gu Y, Lin J, Ding X. Identification and validation of a Metabolism-Related Prognostic Signature Associated with M2 macrophage infiltration in gastric Cancer. Int J Mol Sci 2023, 24(13). 10.3390/ijms241310625 PMC10342140 37445803

[14] Kopeina GS, Zhivotovsky B (2022). Programmed cell death: past, present and future. Biochem Biophys Res Commun.

[15] Wang Y, Yin B, Li D, Wang G, Han X, Sun X (2018). GSDME mediates caspase-3-dependent pyroptosis in gastric cancer. Biochem Biophys Res Commun.

[16] Yang Z, Zou S, Zhang Y, Zhang J, Zhang P, Xiao L, Xie Y, Meng M, Feng J, Kang L (2023). ACTL6A protects gastric cancer cells against ferroptosis through induction of glutathione synthesis. Nat Commun.

[17] Wang R, Xu K, Chen Q, Hu Q, Zhang J, Guan X (2023). Cuproptosis engages in c-Myc-mediated breast cancer stemness. J Translational Med.

[18] Liu X, Nie L, Zhang Y, Yan Y, Wang C, Colic M, Olszewski K, Horbath A, Chen X, Lei G (2023). Actin cytoskeleton vulnerability to disulfide stress mediates disulfidptosis. Nat Cell Biol.

[19] Hanahan D (2022). Hallmarks of Cancer: New dimensions. Cancer Discov.

[20] Kumar V, Ramnarayanan K, Sundar R, Padmanabhan N, Srivastava S, Koiwa M, Yasuda T, Koh V, Huang KK, Tay ST (2022). Single-cell atlas of Lineage States, Tumor Microenvironment, and subtype-specific expression programs in gastric Cancer. Cancer Discov.

[21] Goldman MJ, Craft B, Hastie M, Repečka K, McDade F, Kamath A, Banerjee A, Luo Y, Rogers D, Brooks AN (2020). Visualizing and interpreting cancer genomics data via the Xena platform. Nat Biotechnol.

[22] Cristescu R, Lee J, Nebozhyn M, Kim KM, Ting JC, Wong SS, Liu J, Yue YG, Wang J, Yu K (2015). Molecular analysis of gastric cancer identifies subtypes associated with distinct clinical outcomes. Nat Med.

[23] Ianevski A, Giri AK, Aittokallio T (2022). Fully-automated and ultra-fast cell-type identification using specific marker combinations from single-cell transcriptomic data. Nat Commun.

[24] Kanehisa M, Goto S (2000). KEGG: kyoto encyclopedia of genes and genomes. Nucleic Acids Res.

[25] Gene Ontology Consortium (2015). Going forward. Nucleic Acids Res.

[26] Steen CB, Liu CL, Alizadeh AA, Newman AM (2020). Profiling cell type abundance and expression in bulk tissues with CIBERSORTx. Methods Mol Biol.

[27] Colaprico A, Silva TC, Olsen C, Garofano L, Cava C, Garolini D, Sabedot TS, Malta TM, Pagnotta SM, Castiglioni I (2016). TCGAbiolinks: an R/Bioconductor package for integrative analysis of TCGA data. Nucleic Acids Res.

[28] Li J, Yang P, Chen F, Tan Y, Huang C, Shen H, Peng C, Feng Y, Sun Y (2021). Hypoxic colorectal cancer-derived extracellular vesicles deliver microRNA-361-3p to facilitate cell proliferation by targeting TRAF3 via the noncanonical NF-κB pathways. Clin Translational Med.

[29] Li J, Peng W, Yang P, Chen R, Gu Q, Qian W, Ji D, Wang Q, Zhang Z, Tang J (2020). MicroRNA-1224-5p inhibits metastasis and epithelial-mesenchymal transition in Colorectal Cancer by Targeting SP1-Mediated NF-κB signaling pathways. Front Oncol.

[30] Zhao S, Wang L, Ding W, Ye B, Cheng C, Shao J, Liu J, Zhou H (2023). Crosstalk of disulfidptosis-related subtypes, establishment of a prognostic signature and immune infiltration characteristics in bladder cancer based on a machine learning survival framework. Front Endocrinol.

[31] Stockwell BR (2022). Ferroptosis turns 10: emerging mechanisms, physiological functions, and therapeutic applications. Cell.

[32] He J, Wang X, Chen K, Zhang M, Wang J (2022). The amino acid transporter SLC7A11-mediated crosstalk implicated in cancer therapy and the tumor microenvironment. Biochem Pharmacol.

[33] Thrift AP, El-Serag HB (2020). Burden of gastric Cancer. Clin Gastroenterol Hepatology: Official Clin Pract J Am Gastroenterological Association.

[34] Cao W, Chen HD, Yu YW, Li N, Chen WQ (2021). Changing profiles of cancer burden worldwide and in China: a secondary analysis of the global cancer statistics 2020. Chin Med J.

[35] Luo Q, Zheng N, Jiang L, Wang T, Zhang P, Liu Y, Zheng P, Wang W, Xie G, Chen L (2020). Lipid accumulation in macrophages confers protumorigenic polarization and immunity in gastric cancer. Cancer Sci.

[36] Mehla K, Singh PK (2019). Metabolic regulation of macrophage polarization in Cancer. Trends cancer.

[37] Vitale I, Manic G, Coussens LM, Kroemer G, Galluzzi L (2019). Macrophages and metabolism in the Tumor Microenvironment. Cell Metabol.

[38] Qi C, Ma J, Sun J, Wu X, Ding J (2023). The role of molecular subtypes and immune infiltration characteristics based on disulfidptosis-associated genes in lung adenocarcinoma. Aging.

[39] Wang X, Lin J, Li Z, Wang M (2023). In what area of biology has a new type of cell death been discovered?. Biochim et Biophys acta Reviews cancer.

[40] Wang Z, Chen X, Zhang J, Chen X, Peng J, Huang W (2023). Based on disulfidptosis-related glycolytic genes to construct a signature for predicting prognosis and immune infiltration analysis of hepatocellular carcinoma. Front Immunol.

[41] Wang T, Guo K, Zhang D, Wang H, Yin J, Cui H, Wu W (2023). Disulfidptosis classification of hepatocellular carcinoma reveals correlation with clinical prognosis and immune profile. Int Immunopharmacol.

[42] Xu K, Zhang Y, Yan Z, Wang Y, Li Y, Qiu Q, Du Y, Chen Z, Liu X (2023). Identification of disulfidptosis related subtypes, characterization of tumor microenvironment infiltration, and development of DRG prognostic prediction model in RCC, in which MSH3 is a key gene during disulfidptosis. Front Immunol.

[43] Chen H, Yang W, Li Y, Ma L, Ji Z (2023). Leveraging a disulfidptosis-based signature to improve the survival and drug sensitivity of bladder cancer patients. Front Immunol.

[44] Ni L, Yang H, Wu X, Zhou K, Wang S (2023). The expression and prognostic value of disulfidptosis progress in lung adenocarcinoma. Aging.

[45] Deng G, Zhang X, Chen Y, Liang S, Liu S, Yu Z, Lü M (2023). Single-cell transcriptome sequencing reveals heterogeneity of gastric cancer: progress and prospects. Front Oncol.

[46] Wang X, Almet AA, Nie Q (2023). The promising application of cell-cell interaction analysis in cancer from single-cell and spatial transcriptomics. Sem Cancer Biol.

[47] Mushti SL, Mulkey F, Sridhara R (2018). Evaluation of overall response rate and progression-free survival as potential surrogate endpoints for overall survival in immunotherapy trials. Clin cancer Research: Official J Am Association Cancer Res.

[48] Janjigian YY, Shitara K, Moehler M, Garrido M, Salman P, Shen L, Wyrwicz L, Yamaguchi K, Skoczylas T, Campos Bragagnoli A (2021). First-line nivolumab plus chemotherapy versus chemotherapy alone for advanced gastric, gastro-oesophageal junction, and oesophageal adenocarcinoma (CheckMate 649): a randomised, open-label, phase 3 trial. Lancet (London England).

