# Supplementary figures and images for: Integrated analysis of disulfidptosis-related immune genes signature to boost the efficacy of prognostic prediction in gastric cancer
Source: Cancer Cell Int. 2024 Mar 25;24:112. doi: 10.1186/s12935-024-03294-5 (PMC10962090; doi:10.1186/s12935-024-03294-5)

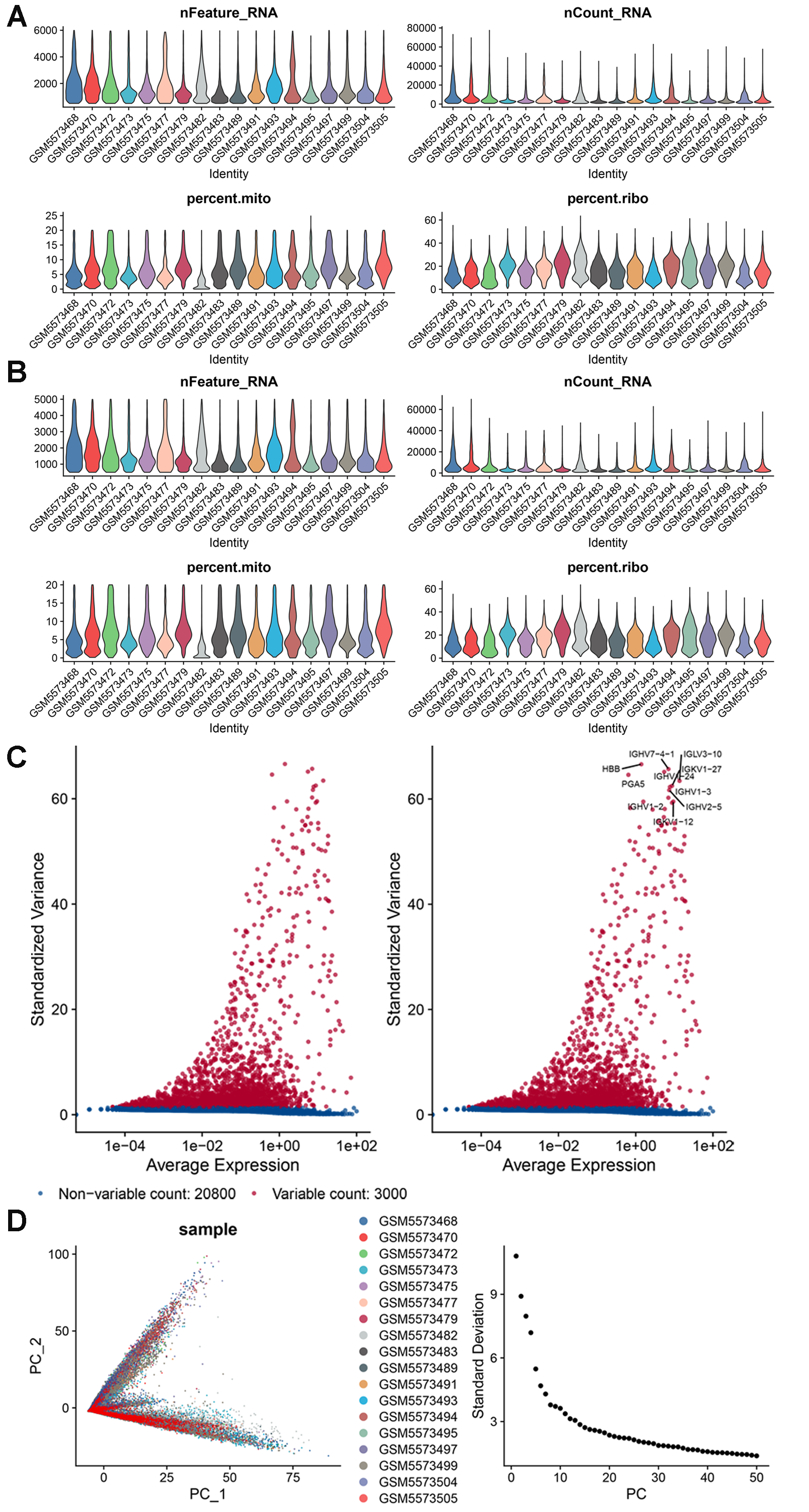

Supplement: Supplementary file 1 — Supplementary Material 1 [file 12935_2024_3294_MOESM1_ESM.tiff]

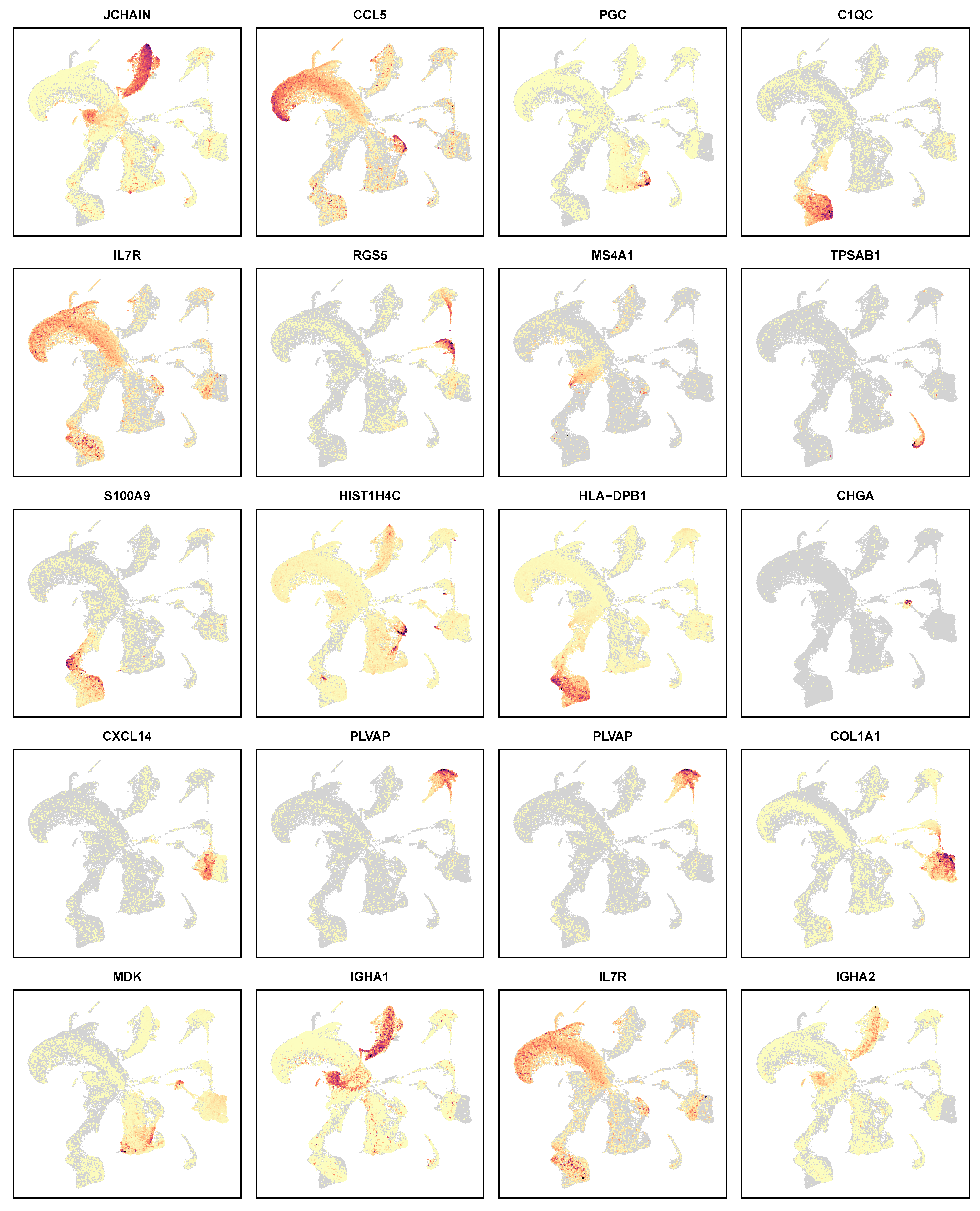

Supplement: Supplementary file 2 — Supplementary Material 2 [file 12935_2024_3294_MOESM2_ESM.tiff]
