# Supplementary material for: Integrated analysis of disulfidptosis-related immune genes signature to boost the efficacy of prognostic prediction in gastric cancer
Source: Cancer Cell Int. 2024 Mar 25;24:112. doi: 10.1186/s12935-024-03294-5 (PMC10962090; doi:10.1186/s12935-024-03294-5)
Supplement: Supplementary file 3 — Supplementary Material 3 [file 12935_2024_3294_MOESM3_ESM.docx]

**Gastric Cancer Dynamic Nomogram User Guide**

**Introduction**

The Gastric Cancer Dynamic Nomogram is an online survival analysis tool designed for patients with gastric cancer. It calculates the probability of survival based on clinical features such as age, gender, tumor staging, and risk level, and dynamically displays the survival curve. This tool aids physicians and patients in better understanding prognostic outcomes.

**Getting Started**

**Inputting Patient Information**

1. **Age**:
   - Adjust the slider to select the patient's age.
   - The slider ranges from 35 to 90 years old.
2. **Gender**:
   - Choose the patient's gender from the dropdown menu (Male or Female).
3. **Pathologic Stage**:
   - Select the patient's pathological stage, which classifies the biological characteristics of the tumor, such as size and invasion.
4. **T Stage**:
   - Indicates the size of the tumor and its local spread. Choose the appropriate option from T1 to T4.
5. **N Stage**:
   - Indicates whether the tumor has spread to nearby lymph nodes. N0 means no spread, and N1 to N3 indicate increasing levels of spread.
6. **M Stage**:
   - Indicates whether the tumor has metastasized to distant sites. M0 means no metastasis, and M1 indicates metastasis.
7. **Risk Level**:
   - Select a risk level based on the patient's RNA-seq information, usually assessed by a physician.

**Predicting Survival Probability**

- **Predict Button**:
  - After entering all necessary information, click the "Predict" button to calculate the survival probability.
- **Survival Plot**:
  - The prediction results will be displayed as a survival curve in the right-hand area. The blue line represents the expected survival probability calculated based on the patient's information entered.

**Interpreting Results**

- **Survival Plot**:
  - This graph shows the survival probability over the follow-up time.
- **Predicted Survival**:
  - If you wish to see the survival probability at a specific time point, use the slider below to select the time (futime). The survival probability at this time point will be calculated and displayed upon clicking "Predict."
- **Numerical Summary**:
  - Provides specific values of survival probabilities at different follow-up times.
- **Model Summary**:
  - Offers detailed statistical information about the predictive model used, including accuracy and reliability metrics.

**Advanced Features**

- **Alpha Blending (Transparency Adjustment)**:
  - If you need to adjust the transparency of the survival curve, check the "Alpha blending" box and use the slider to modify.

**Exiting the Tool**

- **Quit Button**:
  - When you have finished your analysis and need to exit the tool, click the "Quit" button.

**Important Notes**

- The predictions provided by this tool should not be used as the sole basis for decision-making. Consult a medical professional when making significant medical decisions.
- Survival probability predictions are based on statistical models and carry a certain level of uncertainty. Always consider the context and clinical expertise when interpreting these results.
